# NOTCH3 and Pulmonary Arterial Hypertension

**DOI:** 10.3390/ijms25116248

**Published:** 2024-06-06

**Authors:** Nolan M. Winicki, Cristian Puerta, Casandra E. Besse, Yu Zhang, Patricia A. Thistlethwaite

**Affiliations:** Division of Cardiothoracic Surgery, University of California, 9300 Campus Point Drive, La Jolla, San Diego, CA 92037-7892, USA

**Keywords:** NOTCH3, pulmonary arterial hypertension, JAG-1, DLL-4, PAH, HES-5, Notch3

## Abstract

NOTCH3 receptor signaling has been linked to the regulation of smooth muscle cell proliferation and the maintenance of smooth muscle cells in an undifferentiated state. Pulmonary arterial hypertension (World Health Organization Group 1 idiopathic disease: PAH) is a fatal disease characterized clinically by elevated pulmonary vascular resistance caused by extensive vascular smooth muscle cell proliferation, perivascular inflammation, and asymmetric neointimal hyperplasia in precapillary pulmonary arteries. In this review, a detailed overview of the specific role of NOTCH3 signaling in PAH, including its mechanisms of activation by a select ligand, downstream signaling effectors, and physiologic effects within the pulmonary vascular tree, is provided. Animal models showing the importance of the NOTCH3 pathway in clinical PAH will be discussed. New drugs and biologics that inhibit NOTCH3 signaling and reverse this deadly disease are highlighted.

## 1. Introduction

Pulmonary arterial hypertension (PAH: World Health Organization (WHO) Group I idiopathic disease) is defined by sustained elevation of pulmonary arterial pressure (PAP) and pulmonary vascular resistance (PVR), leading to right ventricular failure and death [1,2]. At the cellular level, PAH is characterized by increased vascular smooth muscle cells (vSMCs) resulting in medial hyperplasia [3], neointimal hyperplasia [4,5], and perivascular inflammation [6] in small pulmonary arteries. Multiple processes may contribute to the pathology of PAH, including specific genetic mutations, pulmonary endothelial cell injury, immune perturbations, and abnormal proliferative cues. In specific, there is now substantial evidence that the NOTCH3 pathway and its downstream mediators play an important role in the development of PAH [4,7,8]. Elucidating the molecular mechanisms underlying NOTCH3 signaling may provide valuable insights into the pathogenesis of PAH and potentially lead to the development of novel therapeutic interventions.

Therefore, the rationale for this review stems from recent surges in research focusing on the NOTCH3 pathway, highlighting its role not only in the normal development and function of the lung but also in the pathophysiology of PAH. This review aims to consolidate the current knowledge of the NOTCH3 pathway, elucidate its specific contributions to PAH, and explore potential therapeutic targets. In doing so, it seeks to provide a comprehensive resource for researchers and clinicians alike, foster a deeper understanding of PAH pathogenesis, and guide future research and clinical strategies.

This review is structured to first provide an overview of the NOTCH3 signaling pathway, discussing its biological functions, role in vascular biology, and evidence linking it to PAH. The following text highlights key preclinical and clinical studies that have shaped the understanding of NOTCH3 signaling in PAH. Furthermore, it will discuss the therapeutic implications of targeting the NOTCH3 pathway, review translational experiments, and explore future possibilities.

By the end of this discussion, readers will gain a clear understanding of the critical role of the NOTCH3 pathway in PAH, appreciating the complexity of its regulation and the potential for therapeutic intervention. This review will also identify gaps in the current knowledge and suggest directions for future research, potentially guiding the next steps in PAH treatment and management.

## 2. NOTCH3 and Pulmonary Arterial Hypertension

NOTCH3 is one of four mammalian NOTCH proteins (NOTCH 1–4), which acts as a transmembrane signaling receptor in developmental patterning, cell fate decisions, and regulation of cell survival and proliferation [9]. The NOTCH3 receptor is composed of a large 210 kDa extracellular domain (ECD) that is mostly tandem repeats of epidermal growth factor (EGF), a transmembrane portion, and a smaller 97 kDa intracellular domain (ICD) [10,11]. Several lines of evidence have shown that Notch cleavage occurs when a membrane-bound Notch ligand binds to the ECD and mechanically pulls it toward the ligand-presenting cell, opening up the cleavage sites for Notch receptor proteolysis [12]. After Notch cleavage, the ligand is endocytosed into the ligand-presenting cell, which may be the receptor-expressing cell itself (cis-activation or autocrine activation) or the adjacent cell (trans-activation or paracrine activation), and recycled for presentation on the cell surface [12,13,14].

**Figure 1:**
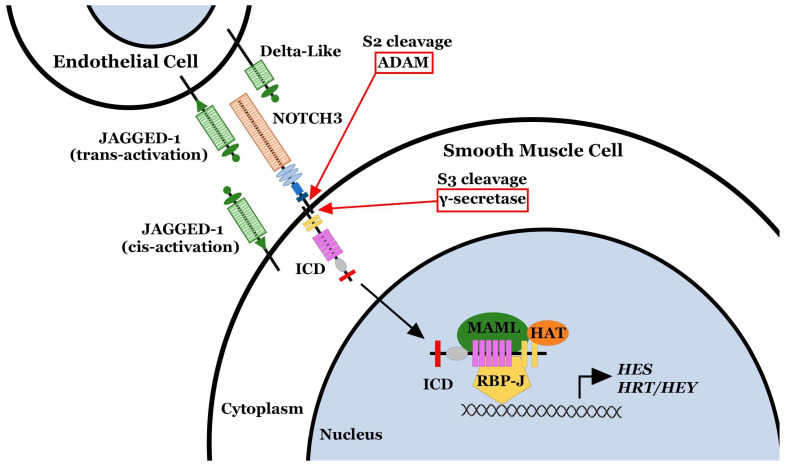
NOTCH3 is activated by the binding of the JAGGED-1 ligand, stimulating the proteolytic S2 cleavage by ADAM of the NOTCH3 extracellular domain (ECD) and intracellular domain (ICD). Then, S3 cleavage by γ-secretase liberates the ICD into the cytoplasm. The ICD translocates to the nucleus and binds to the RBP-J transcription complex and functions as a transcriptional enhancer of *Hairy/Enhancer of Split* (*HES*) and *Hairy/Enhancer of Split-related* (*HRT/HEY*) genes.

The NOTCH3 receptor is uniquely expressed on vSMCs from small resistance pulmonary arteries (<1500 μm diameter in the human lung and <500 μm in diameter in the rodent lung) [8]. By lineage tracing, Notch3 has been shown to be the dominant marker for neointimal cells obstructing pulmonary hypertensive vessels [4]. Within the pulmonary vasculature, NOTCH3 signaling is initiated by the binding of the selective NOTCH ligand, JAGGED-1 (JAG-1) to the receptor, stimulating cleavage of the receptor into two peptides: an intracellular domain (NOTCH3 ICD) and an extracellular domain (NOTCH3 ECD) (Figure 1) [15]. The NOTCH3 ICD translocates to the nucleus and functions as a transcriptional enhancer of *Hairy/Enhancer of Split* (*HES*) and *Hairy/Enhancer of Split-related* (*HRT/HEY*) genes, whose protein products are modulators of the transcription of genes involved in cellular homeostasis and proliferation (Figure 1) [16,17]. Notably, the specific downstream mediator of NOTCH3 ICD signaling in the pulmonary vasculature, HES-5, has been linked to vSMC proliferation and anti-apoptosis, as well as a shift to an undifferentiated vSMC phenotype [8,18]. In contrast, the fate of the NOTCH3 ECD is less well known in the pulmonary vasculature. Studies in Drosophila and mammalian cell lines have demonstrated that after cleavage of the Notch receptor, the Notch ECD is liberated from the plasma membrane and released into the interstitium and blood, while the Notch ligand (Jag-1) is internalized into the ligand-presenting cell [19]. In other circumstances, the entire Notch ECD/Jag-1 complex may be endocytosed and internalized into the ligand-presenting cell, with Jag-1 recycled back to the cell surface and Notch ECD degraded [12].

## 3. NOTCH3 Is a Marker of PAH Disease Severity

**Figure 2:**
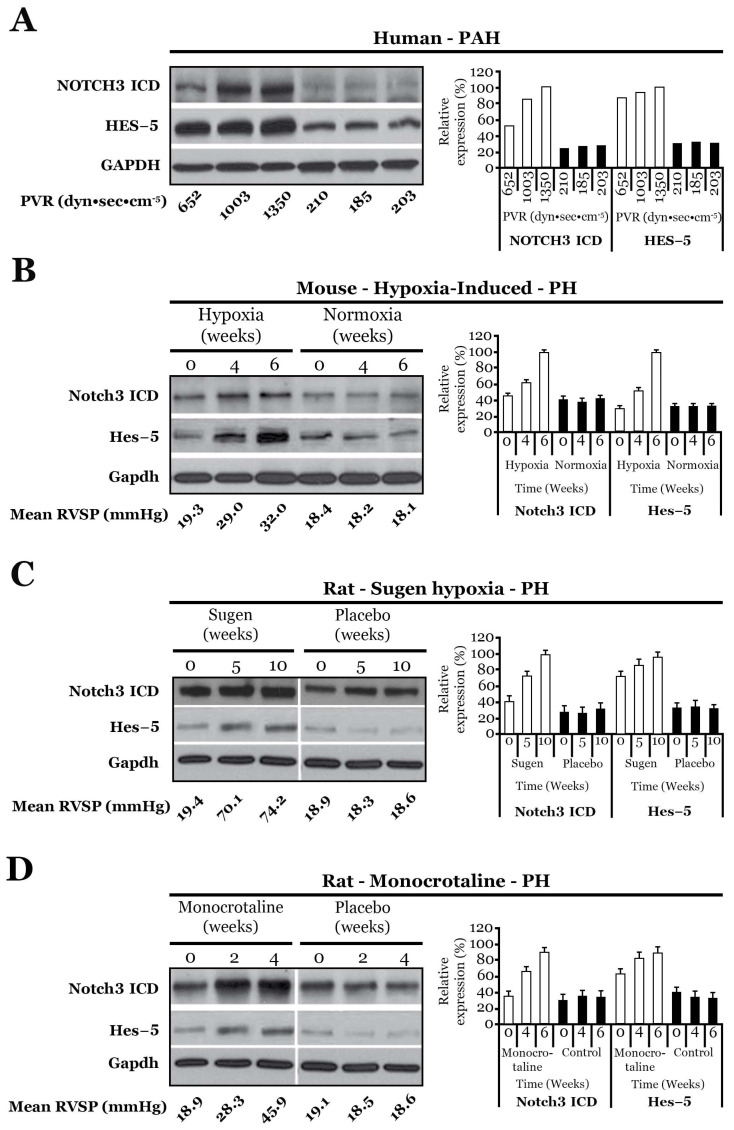
(**A**) **Left:** Western blot analysis of NOTCH3 ICD and HES-5 relative to GAPDH in the lungs of subjects with varying severities of PAH and control individuals. ICD, intracellular domain. PVR, pulmonary vascular resistance. **Right:** Relative expression values obtained by densitometry of NOTCH3 ICD or HES-5 protein normalized to GAPDH (n = 1 for each PVR listed). (**B**) **Left:** Western blot analysis of Notch3 ICD and Hes-5 relative to Gapdh from mouse lungs during the development of hypoxia-induced pulmonary hypertension, compared to normoxic animals. **Right:** Relative expression values obtained by densitometry of Notch3 ICD or Hes-5 proteins normalized to Gapdh (n = 20 animals for each time point). RVSP, right ventricular systolic pressure (mmHg). (**C**) **Left:** Western blot analysis of Notch3 ICD and Hes-5 relative to Gapdh from rat lungs during the development of Sugen-induced pulmonary hypertension, compared to control animals (For (**B**–**D**), four measurements of RSVP were taken over a 10-min period and averaged). **Right:** Relative expression values obtained by densitometry of Notch3 ICD or Hes-5 protein normalized to Gapdh (n = 20 animals for each time point). (**D**) **Left:** Western blot analysis of Notch3 ICD and Hes-5 relative to Gapdh from rat lungs during the development of monocrotaline-induced pulmonary hypertension, compared to control animals. **Right:** Relative expression values obtained by densitometry of Notch3 ICD or Hes-5 protein normalized to Gapdh (n = 20 animals for each time point). Figure modified and adapted from previous publication [8].

There are three well-known rodent models of pulmonary hypertension (PH) [20]. A mouse placed in 10% oxygen will develop vascular reactivity as well as vascular changes of PH, including medial hyperplasia, in approximately four weeks [21]. Rats injected with the Flk-1 receptor tyrosine kinase (also known as Vegfr2) inhibitor, SU5416 (Sugen), followed by housing in 10% oxygen develop PH that most recapitulates human PAH, with medial hyperplasia and neointima formation, luminal narrowing of small precapillary pulmonary arteries, and the development of plexiform lesions [22]. Morphologically, plexiform lesions show excessive cellular occlusion of the lumina of small pulmonary vessels with adjacent blind off-shoots of occluded endothelial tubes [23]. Rats intraperitoneally injected with the pyrrolizidine alkaloid compound, monocrotaline, will develop PH within four weeks and ultimately die of this disease [24]. Monocrotaline-treated rodents also have a higher propensity to develop pulmonary fibrosis and adenocarcinomas of the lung compared to age-matched, untreated littermates [24]. Each of these three rodent models show progressive worsening of clinical and pathologic disease over time (Figure 2) [8,14].

The lungs of mice with hypoxia-induced PH had elevated levels of *Notch3* mRNA and Notch3 ICD protein compared to the lungs of control littermates kept under normoxic conditions [8]. Higher levels of *Notch3* mRNA were also observed in the lungs, brain, heart, and kidney of pulmonary hypertensive, hypoxic mice than in non-pulmonary hypertensive, normoxic mice. However, in mice with hypoxia-induced PH, minimal levels of Notch3 ICD protein were detected in most organs, while the lung levels of Notch3 ICD protein were more than triple that of controls. This suggested that Notch3 signaling, and cleavage may be associated with the pathogenesis of PH. Similarly, rats with monocrotaline-induced PH demonstrated progressive elevation in steady-state levels of *Notch3* mRNA and ICD protein within the lungs compared to controls throughout disease progression [8]. In contrast, no difference was found in the expression of Notch1, 2, or 4 in the lungs of normotensive rats versus the lungs of monocrotaline-induced PH rats [8]. In the Sugen-hypoxia model of rat PH, there are marked elevations in Notch3 ICD in the lungs of rats with PH [8]. In all three rodent models of PH, worsening severity of disease, as measured by right ventricular systolic pressure (RVSP), systolic PAP, the right ventricular weight compared to the left ventricle and septum ratio, an index of right ventricular hypertrophy (Fulton index) [25], vessel pruning on angiography, and lung pathology correlated with the amount of Notch3 ICD within the lungs (Figure 2) [8,14]. By immunohistochemistry, Notch3 ICD protein localized to the media of small precapillary pulmonary arteries in all three rodent models of disease [8,14].

Humans undergoing lung transplantation for end-stage PAH have markedly elevated levels of *NOTCH3* mRNA and ICD protein in the lung compared to the lung tissue of healthy age-matched controls (Figure 2) [8]. Specifically, there was a linear relationship between the degree of PAH severity as measured by PVR, mean PAP (mPAP), and tricuspid regurgitant velocity (TVR) and NOTCH3 ICD protein levels in the lung. Individuals with normal PVR (<250 dynes·s·cm^−5^) had minimal levels of NOTCH3 ICD in their lungs, while patients with supra-systemic PVRs had massive elevation of NOTCH3 ICD in their lungs (Figure 2). Immunohistochemical staining revealed that NOTCH3 ICD was localized to small precapillary pulmonary arteries measuring <1500 μm in diameter [8].

## 4. Activation of NOTCH3 via JAG-1 Increases the Proliferation of Small Pulmonary Artery Smooth Muscle Cells

Elevations in the NOTCH3 ligand, JAG-1, influence NOTCH3 signaling and proliferation of human small pulmonary artery smooth muscle cells (sPASMCs: isolated from vessels < 1500 μm in diameter in humans and <500 μm in diameter in rodents) [26]. Subcultured sPASMCs from healthy, non-PAH individuals, infected with a *JAG1* lentivirus to stimulate constitutive NOTCH3 signaling, demonstrated increased NOTCH3 ICD protein compared to the control, empty vector–transduced sPASMCs [8]. Constitutive JAG-1 expression induced a more rapid growth rate, as determined by ^3^[H]leucine incorporation and cell count of sPASMCs [8]. In contrast, inhibition of the JAG-1/NOTCH3 signaling cascade by transfection of *JAG1*-specific short hairpin RNA (shRNA) into non-confluent sPASMCs resulted in a significant decrease in NOTCH3 ICD, as demonstrated by Western blotting, as well as reduced ^3^[H]leucine incorporation and sPASMC cell count [8].

Conversely, non-confluent sPASMCs infected with a *Delta-like ligand 4 (DLL4)* lentivirus showed a reduction in NOTCH3 ICD protein levels and exhibited stunted growth compared to empty vector–transduced sPASMCs [8]. In particular, the presence of DLL-4 in the same cell (in cis) prevented the cleavage of NOTCH3, indicating that DLL-4 may be competing with JAG-1 for binding to NOTCH3 [27]. This interaction was further defined when transfection of *DLL4* shRNA into sPASMCs resulted in moderately increased NOTCH3 cleavage and elevated cell growth, suggesting that knockdown of *DLL4* may permit more unopposed JAG-1 binding to NOTCH3 [8].

## 5. JAG-1 and NOTCH3 Signaling Is Constitutive in PAH

**Figure 3:**
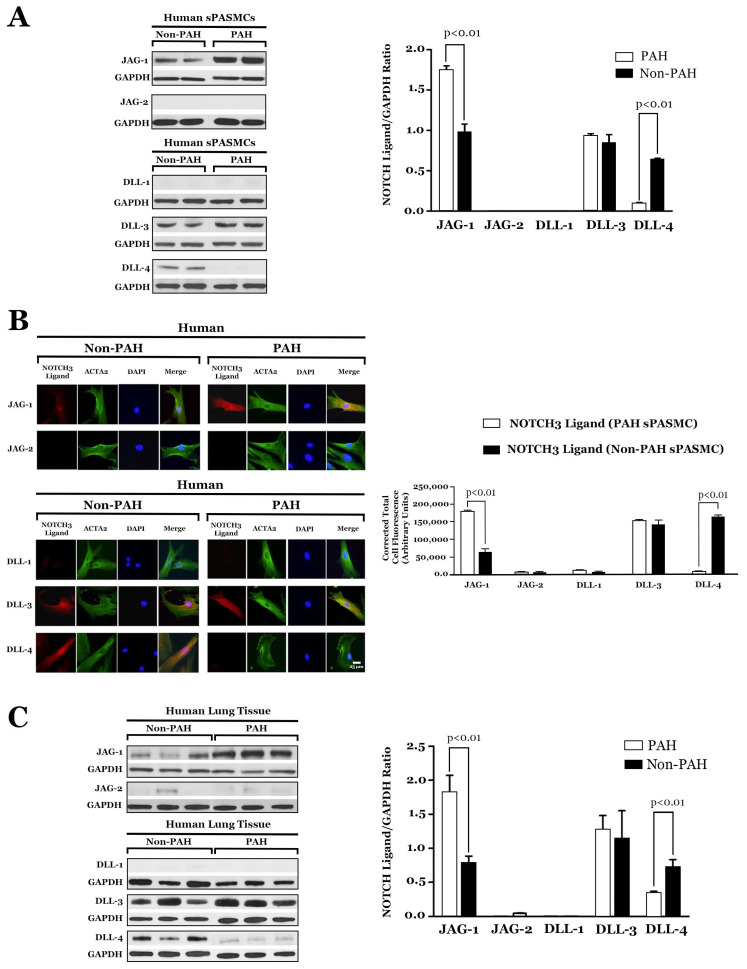
(**A**) **Left:** Western blot analysis of NOTCH ligands relative to GAPDH in subcultured sPASMCs from PAH and non-PAH individuals. **Right:** Relative expression values obtained by densitometry of NOTCH3 ligand proteins normalized to GAPDH (n = 10 sPASMC subcultures per group). (**B**) **Left:** NOTCH3 ligand (red) and α-smooth muscle actin (ACTA2; green) immunofluorescence staining of human PAH and non-PAH-subcultured sPASMCs. Nuclei were counterstained with 4′,6-diamidino-2-phenylindole (DAPI) (blue). Scale bar, 25 μm. **Right:** Relative fluorescence values obtained by densitometry of NOTCH3 ligands normalized to background readings (n = 10 representative cells for each NOTCH3 ligand). (**C**) **Left:** Western blot analysis of NOTCH ligands relative to GAPDH in lung tissue from three different individuals. **Right:** Relative expression values obtained by densitometry of NOTCH3 ligand proteins normalized to GAPDH (n = 20 lung samples from 20 patients in each group). Figure modified and adapted from previous publication [8].

JAG-1 serves as a selective marker for PAH in sPASMCs in the human lung [14]. Western blotting and semi-quantitative immunofluorescence staining confirmed higher amounts of JAG-1 and lower amounts of DLL-4 in sPASMCs from PAH lung tissue compared to age- and sex-matched control cells from normotensive tissue (Figure 3) [8]. Lung biopsies from individuals with PAH have shown elevated amounts of JAG-1 protein and reduced levels of DLL-4 protein in pulmonary hypertensive lung tissue compared to normotensive lung specimens by Western blotting (Figure 3) [8]. In contrast, in lung tissue from non-PAH patients, DLL-4 protein predominated, with minimal JAG-1 protein seen by Western blotting (Figure 3) [8]. These results support the proliferative role of JAG-1 and the imbalance between JAG-1 and DLL-4 in the pathogenesis of idiopathic PAH [8,28].

## 6. Role of DLL-4 in the Development of PAH

Recent clinical trials investigating DLL-4 inhibitors, such as Demcizumab [29,30], Enoticumab [31], ABL001 [32], Navicixizumab [33], and Dipacimab [34], for the treatment of oncologic malignancies, have shown that these drugs induce PAH as an unanticipated, deleterious side effect [30,34,35,36]. The risk of PAH correlated with the dosage and duration of DLL-4 inhibitor treatment. These clinical data support previous translational experiments which showed that reduced expression of DLL-4 in sPASMCs with shRNA (unopposed JAG-1 signaling) resulted in increased sPASMC proliferation [8]. A separate murine study has also demonstrated that continuous administration of a DLL-4 neutralizing antibody promoted the development of PH by impairing endothelial cell barrier function and increased immune cell infiltration [37]. Additionally, the findings that levels of DLL-4 were lower in human PAH lung compared to human normotensive lung tissue further suggest that the interplay between JAG-1 and DLL-4 in NOTCH3 activation is critical to the homeostasis in the pulmonary vasculature. Thus, unopposed constitutive JAG-1 protein expression, without DLL-4 competition in patients treated with DLL-4 inhibitors, is presumed to induce vSMC hyperplasia in small precapillary pulmonary arteries with luminal narrowing and occlusion, resulting in clinical disease with elevated PVR.

## 7. HES-5, a Downstream Effector of NOTCH3 Signaling, Drives the Pulmonary Hypertensive Phenotype

NOTCH3 is known to stimulate the downstream transcription of two major classes of genes, termed *HES* and *HRT* (also known as *HEY*). Specifically, HES-5 has been found to be increased in human PAH lungs and rodent PH lungs in three rodent models of disease [8,14,38]. Constitutive levels of HES-5 in human and rodent lung tissues correlated with disease severity, as measured by PVR in humans and RVSP in rodents [8]. Notably, there was no difference in the expression of other *Notch3* target genes, *Hes1*, *Hes7*, *Hrt1*, *Hrt2*, and *Hrt3*, in the lungs of pulmonary hypertensive versus normotensive humans and rodents [8]. These results suggested that HES-5, the downstream effector of NOTCH3 signaling, is a specific marker for the severity of PAH in humans and PH in rodents [8,14,38].

To understand the effects of constitutive HES-5 expression on sPASMCs, a series of experiments were performed and published [8]. First, subcultured non-PAH human sPASMCs infected with a *Notch3* ICD adenovirus resulted in increased NOTCH3 ICD and HES-5 protein expression compared to the control, *lacZ* adenovirus-transduced sPASMCs [8]. Constitutive, high-level HES-5 expression in sPASMCs demonstrated a significantly increased growth rate and increased ^3^[H]leucine incorporation rate at preconfluence [8]. Transfection of non-PAH sPASMCs constitutively expressing NOTCH3 ICD with *HES5*-specific small interfering RNA (*HES5* siRNA) resulted in significantly decreased HES-5 expression, with reduced ^3^[H]leucine incorporation and cellular proliferation compared to non-PAH sPASMCs transfected with a control (scrambled) oligonucleotide [8].

Second, in human and mouse lungs, HES-5 expression was restricted to vSMCs in the small precapillary pulmonary arteries [8]. HES-5 staining was localized in the media of small precapillary pulmonary arteries, with sporadic staining in the neointimal vessels [8]. NOTCH3 ICD and HES-5 staining was not detected in the pulmonary veins and venules [8,14]. The above in vitro and in vivo results suggested that the NOTCH3–HES-5 signaling pathway is constitutively active in the sPASMCs of the media of small precapillary pulmonary arteries in the PAH lung and that high levels of HES-5 protein were associated with the development of medial hyperplasia [8].

### Proliferation of PAH sPASMCs Is Dependent on NOTCH3-HES-5

The NOTCH3–HES-5 signaling pathway was then extended to the pathogenesis of human PAH by the comparison of primary subcultured sPASMCs from ten human subjects with PAH to ten human subjects without PAH [8]. Notably, sPASMCs derived from the lungs of individuals with PAH exhibited shorter doubling times and higher rates of ^3^[H]leucine incorporation compared to sPASMCs derived from non-PAH lungs [8]. The subcultured sPASMCs from the pulmonary hypertensive lung tissues of both humans and mice displayed higher levels of NOTCH3 and HES-5 staining compared to the normotensive age- and sex-matched control lung tissue [8]. Inhibition of the expression of HES-5, by *HES5* siRNA, significantly reduced proliferation and ^3^[H]leucine incorporation in PAH sPASMCs, indicating that NOTCH3 signaling through HES-5 promotes pulmonary arterial medial hyperplasia [8]. siRNA-mediated knockdown of *HES5* in PAH sPASMCs also displayed evidence of increased expression of the vSMC contractile markers MYH11 (encoding myosin heavy chain) and SMTN (encoding smoothelin) [39] compared to untreated or scrambled siRNA-treated PAH sPASMCs [8]. These results demonstrated that the enhanced NOTCH3 signaling through HES-5 seen in PAH sPASMCs contributed to the ability of these cells to proliferate and lose expression of markers of contractile vSMCs [8].

## 8. *Notch3^−/−^* Mice Do Not Develop PH

Previous work has demonstrated that Notch3 signaling is required for the development of hypoxia-induced PH [8]. Specifically, *Notch3*-null mice (with homozygous deletion of *Notch3*, lacking the 2.5 kb of genomic sequence encoding EGF-like repeats 8-12 in the ECD) were compared to wild-type mice [8]. Minimal Hes-5 expression in the lung under both hypoxic (10% oxygen) and normoxic conditions was observed in *Notch3*^−/−^ mice [8]. In contrast, constitutively high levels of Notch3 ICD and Hes-5 proteins were found in the lungs of *Notch3^+/+^* mice that were subjected to four weeks of hypoxia in 10% oxygen [8].

*Notch3*^+/+^ mice manifested progressively elevated RVSP over six weeks of hypoxia, whereas *Notch3*^−/−^ mice did not [8]. The Fulton index minimally changed over the course of hypoxia in *Notch3*^−/−^ mice but significantly increased in *Notch3*^+/+^ mice [8]. After exposure to chronic hypoxia, *Notch3*^+/+^ mice developed excessive small pulmonary artery muscularization and luminal narrowing, consistent with advanced pulmonary hypertension [40], whereas *Notch3*^−/−^ mice had normal-appearing small arteries without muscular thickening [8]. Although measurable pulmonary arterial wall thickness in small pulmonary arteries increased in *Notch3*^+/+^ mice compared to *Notch3*^−/−^ mice, there was no significant difference between the groups in the vessel/alveoli ratios [8]. Furthermore, *Notch3*^−/−^ mice exhibited normal pulmonary angiograms with diffuse vascular blush [8]. Conversely, *Notch3*^+/+^ littermates displayed angiograms indicative of severe small-vessel pruning [8] similar to that seen in human PAH [40].

Further experiments have been performed in *Notch3* knockout and wild-type mice to determine whether pulmonary vasoreactivity was affected by the loss of Notch signaling [8]. Specifically, agonist-mediated vasoconstriction of isolated intrapulmonary small arteries from *Notch3*^−/−^ mice and *Notch3*^+/+^ littermates does not differ significantly after high K+ and prostaglandin F_2α_ treatment [8]. There was also no difference in the relationship between myogenic tone and pulmonary blood flow between *Notch3*^+/+^ and *Notch3*^−/−^ mice, as incremental increases in pulmonary blood flow caused similar augmentation of PAPs in both groups [8]. Under normoxic conditions, *Notch3*^+/+^ and *Notch3*^−/−^ mice demonstrated comparable pulmonary vasoreactivity to vasodilator infusion and had similar baseline measurements for PAP and calculated total PVR [8]. Compared to chronically hypoxic *Notch3*^+/+^ mice, the chronically hypoxic *Notch3*^−/−^ mice had less change in mean PAP and PVR in response to a vasodilator challenge [8], suggesting that PH in these mice was ‘fixed’, due to small-vessel pathological changes, rather than to altered vasoreactivity. Collectively, these results showed that Notch3 signaling is mainly involved in pulmonary vascular remodeling and the pathogenesis of PH, rather than affecting pulmonary vasoreactivity [8].

## 9. Notch3 Inhibition with a γ-Secretase Inhibitor Reverses PH

Cleavage of Notch proteins to ICD and ECD peptides is inhibited by the γ-secretase inhibitor (N-[N-(3, 5-difluorophenacetyl)-l-alanyl]-s-phenylglycinet t-butyl ester) (DAPT) both in vitro and in vivo [41]. Previous work has addressed the hypothesis that administration of DAPT would reverse established hypoxia-induced PH in mice [8]. In these experiments, mice were housed in 10% oxygen for four weeks to induce PH and then treated with daily subcutaneous doses of DAPT or placebo while in the hypoxia chamber for an additional six weeks [8]. DAPT-treated mice displayed diminished levels of Notch3 ICD and Hes-5 in the lungs compared with placebo-treated mice [8]. During the course of treatment, sham-treated mice developed progressive medial thickening of small pulmonary arteries and arterioles [8], consistent with the usual pattern of PH development in hypoxic animals [42], whereas DAPT-treated mice had normal-appearing pulmonary vessels with rarely detected medial thickening or vessel occlusion [8]. Few proliferating vSMCs were observed in the walls of small pulmonary arteries of DAPT-treated mice compared to sham-treated controls, using proliferating cell nuclear antigen immunofluorescence staining [8]. Furthermore, mice treated with DAPT had an increased number of apoptotic cells in the remodeled small pulmonary arteries, as determined by TUNEL staining [8]. These results indicated that the therapeutic effect of DAPT on hypoxia-induced PH involves both antiproliferative and pro-apoptotic effects on sPASMCs [8].

Mice receiving DAPT showed significant reductions in RVSPs relative to systolic blood pressures, as measured by pressure transduction, whereas control mice developed PH with elevated RVSPs [8]. Angiograms performed in chronically hypoxic, DAPT-treated mice revealed a diffuse vascular blush, indicative of a patent distal pulmonary vascular tree [8]. In contrast, angiograms performed in placebo-treated mice displayed blunting of the pulmonary vasculature with a lack of peripheral filing of the arterioles [8]. The ratios of vessels/alveoli were not significantly different between the hypoxic DAPT-treated and placebo-treated mouse lungs [8]. These findings suggest that the vascular pruning observed through angiography in control mice was caused by vessel stenosis and occlusion rather than vessel loss [8]. DAPT-treated mice displayed regression of right ventricular hypertrophy on serial echocardiography [8], further indicating that they were effectively treated for PH [25]. Although gastrointestinal side effects have been reported with other γ-secretase inhibitors [43,44], no overt side effects from DAPT administration were observed with a daily dosing of 10 mg/kg DAPT per body weight [8].

Other studies employing the rat monocrotaline-PH model have demonstrated that DAPT treatment effectively reversed elevations in RVSP, Fulton index, and vascular pathology [45]. Suppressed vascular proliferation and enhanced apoptosis of pulmonary vascular cells was also found after DAPT treatment [8]. Compared to placebo-treated PH rats, there were significantly lower levels of Notch3 ICD in the lungs of the DAPT-treated group than in the placebo-treated group [8]. These above results confirm the ability of DAPT to reverse PH in both murine and rodent models of disease [8].

## 10. Monoclonal Antibody That Blocks JAG-1/NOTCH3 Binding Inhibits NOTCH3 Cleavage

In order to create a selective JAG-1/NOTCH3 inhibitor, a monoclonal antibody (anti-NOTCH3 Ab 28042, AVEO Pharmaceuticals) was created that binds exclusively to NOTCH3 ECD at amino acids 40 to 467 and EGF-like repeats 1 to 11 and blocks autocrine and paracrine JAG-1-induced cleavage of NOTCH3 [14]. Testing with fluorescence-activated cell sorting and surface plasmon resonance analysis demonstrated that anti-NOTCH3 Ab 28042 bound specifically to human and murine NOTCH3 expressed on the cell surface, but not NOTCH1, NOTCH2, and NOTCH4 [14]. Specificity of the monoclonal antibody is critical for the design of biologics for human use, as inhibition of other Notch receptors has been shown to instigate serious side effects due to the systemic expression of Notch1, 2, and 4 in other organ systems in rodents and humans [27,36,46].

In vitro testing was performed to analyze whether anti-NOTCH3 Ab 28042 was specific for JAG-1 binding to NOTCH3, and not for DLL-4 binding to NOTCH3 [14]. In nonconfluent sPASMCs overexpressing JAG-1, the administration of anti-NOTCH3 Ab 28042 significantly reduced NOTCH3 cleavage as well as the levels of HES-5 compared with placebo treatment [14]. In contrast, the administration of anti-NOTCH3 Ab 28042 to nonconfluent sPASMCs overexpressing DLL-4 did not affect NOTCH3 ICD or HES-5 amounts compared to the placebo [14], confirming the specificity of the monoclonal antibody for blocking JAG-1/NOTCH3 interactions only.

Nonconfluent PAH sPASMCs, which are known to constitutively express JAG-1 in an autocrine fashion, were treated with anti-NOTCH3 Ab 28042 and demonstrated a reduction in NOTCH3 ICD and HES-5 proteins, as well as retarded proliferation compared to nonconfluent non-PAH sPASMCs, which do not express JAG-1 [14]. Thus, both JAG-1 overexpression studies in normal sPASMCs and PAH sPASMCs confirmed that the administration of anti-NOTCH3 Ab 28042 displayed a targeted reduction in the NOTCH3-JAG-1 pathway with consequential amelioration of sPASMC proliferation [14].

### 10.1. Anti-NOTCH3 Ab 28042 Treatment Reverses PH in Mice

Subcutaneous administration of the monoclonal antibody anti-NOTCH3 Ab 28042 reversed PH in a hypoxic-PH murine model [14]. Specifically, mice were housed in 10% oxygen for four weeks until they developed clinical and pathologic PH and then were treated with the anti-NOTCH3 Ab 28042 administered subcutaneously three times per week for 12 weeks, while continually housed in 10% oxygen [14]. The levels of Notch3 ICD and Hes-5 were found to be lower in the lungs of the antibody-treated mice compared to those of the placebo-treated mice [14]. The placebo-treated mice displayed progressive medial thickening of their small pulmonary arteries, while the mice treated with anti-NOTCH3 Ab 28042 had normal-appearing pulmonary vessels [14].

Serial echocardiography demonstrated that the administration of anti-NOTCH3 Ab 28042 reversed PH, as evidenced by significant reductions in RVSP and PVR in the treatment group, while the placebo-treated animals continued to develop progressively elevated RVSP and PVR [14]. The antibody-treated mice also displayed regression of RV hypertrophy [14], indicating successful treatment of PH [25]. Pulmonary angiograms conducted on chronically hypoxic, anti-NOTCH3 Ab 28042-treated mice revealed vascular blush throughout their lungs [14], indicating a diffusely patent pulmonary arterial tree [47]. In contrast, chronically hypoxic, placebo-treated control mice demonstrated blunting of the pulmonary vasculature with the absence of peripheral blush on angiography [14]. The vessel/alveoli ratios between the hypoxic antibody-treated and placebo-treated mouse lungs were not significantly different [14]. These results indicated that vascular pruning observed on angiography in control mice was due to vessel occlusion and stenosis rather than vessel loss [14].

While gastrointestinal side effects have been reported with nonspecific NOTCH inhibitor drugs administration in mice [48], no clinical side effects or pathologic changes in organs other than the lungs with anti-NOTCH3 Ab 28042 treatment were observed [14].

### 10.2. Treatment with Anti-NOTCH3 Ab 28042 Reverses PH in Rats

Since the mouse hypoxia model of PH is known to exhibit less vessel thickening compared to other rodent models of PH [49], further experiments were conducted to assess whether treatment with anti-NOTCH3 Ab 28042 would reverse PH in a rat Sugen-hypoxia model of disease [14].

**Figure 4:**
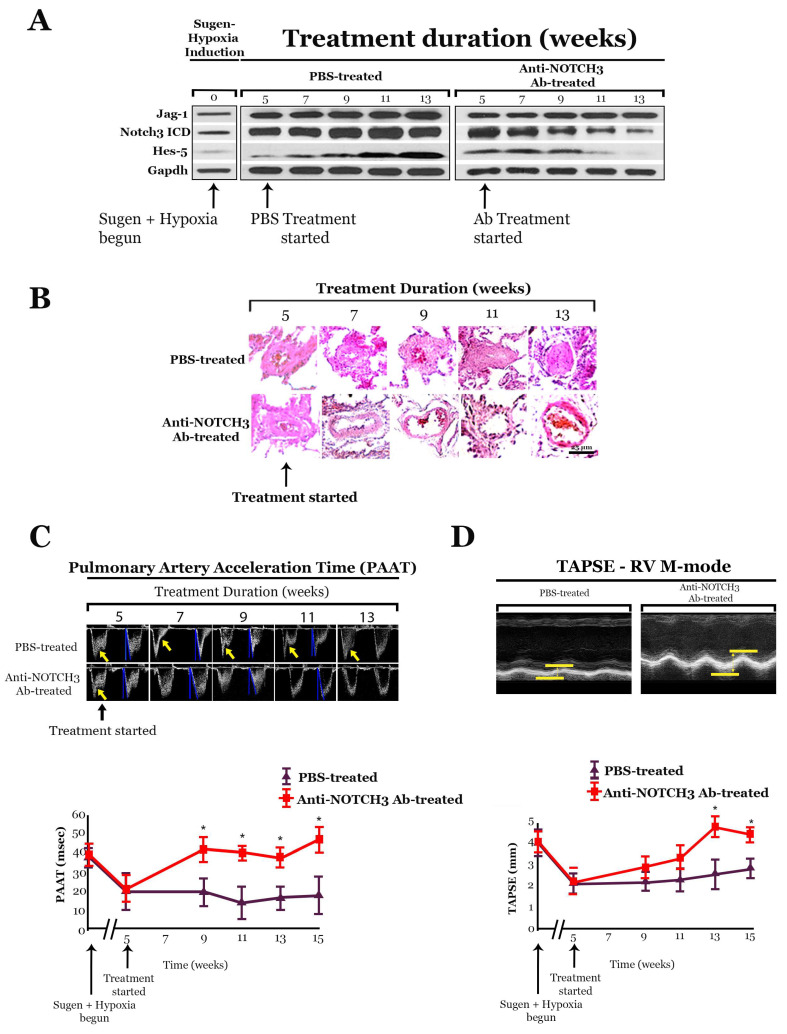

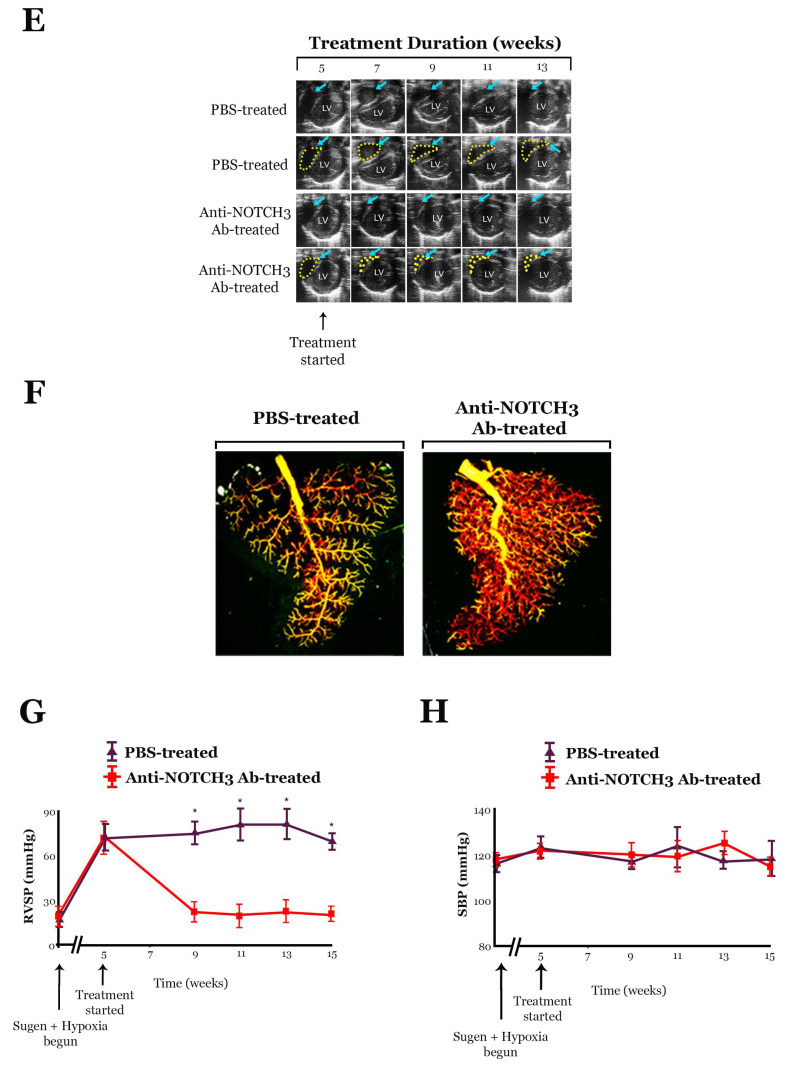
Rats were intraperitoneally injected with Sugen (20 mg/kg) followed by 3 weeks of hypoxia and 2 weeks of normoxia to induce pulmonary hypertension (PH). Subsequently, animals were treated with subcutaneous anti-NOTCH3 Ab 28042 (40 mg/kg) or placebo (PBS) under normoxic conditions for 10 weeks. (**A**) Western blot analysis of Jag-1, Notch3 ICD, and Hes-5 in the lungs of Sugen-hypoxia rats treated with anti-NOTCH3 Ab 28042 or PBS, normalized to Gapdh. (**B**) H&E-stained sections of small pulmonary arteries from lungs of rats at the beginning of treatment and during treatment with anti-NOTCH3 Ab 28042 or PBS. Results are representative sections from five rats per group at each time point. Scale bar, 25 μm. (**C**) **Top:** Representative continuous-wave Doppler signals measured from the right ventricular outflow tract before and during rat treatment with anti-NOTCH3 Ab 28042 compared to controls. Main pulmonary artery blood flow in PBS-treated rats showed shortened acceleration to peak velocity and midsystolic notching (yellow arrows), indicative of elevated pulmonary vascular resistance. **Bottom:** Graph of pulmonary artery acceleration time (PAAT; three measurements per animal per timepoint) in anti-NOTCH3 Ab 28042–treated rats versus controls. (**D**) **Top:** Representative M-mode recordings through the lateral tricuspid annulus, obtained from the apical four-chamber view, from the hearts of rats treated for 10 weeks with anti-NOTCH3 Ab 28042 versus PBS. Tricuspid annular plane systolic excursion (TAPSE) was measured from the end of diastole (lower bar) to the end of systole (top bar). **Bottom:** Graph of TAPSE (three measurements per animal per time point) in anti-NOTCH3 Ab 28042-treated rats (n = 10) versus controls (n = 10). (**E**) Representative parasternal short-axis midventricle echocardiograms of hearts, before and during treatment of rats with anti-NOTCH3 Ab 28042 compared to controls. The right ventricle is marked with blue arrows (rows 1–4) and yellow dotted lines (rows 2 and 4). LV, left ventricle. (**F**) Pulmonary angiograms of PBS-treated and anti-NOTCH3 Ab 28042-treated rats. Results are representative angiograms of the left upper lobe from 10 rats per group after 10 weeks of treatment. (**G**) Average right ventricular systolic pressure (RVSP) in rats before and during treatment with anti-NOTCH3 Ab 28042, compared to PBS-treated controls (15 readings per rat; 10 rats per group at each time point). (**H**) Averaged systolic blood pressure (SBP) in rats treated before and during treatment with anti-NOTCH3 Ab 28042, compared to PBS-treated controls (15 readings per rat; 10 rats per group at each time point). * *p* < 0.05. Figure modified and adapted from previous publication [14].

Similar to the effects in mice, treatment with anti-NOTCH3 Ab 28042 had an effect on the pulmonary vasculature, with the amounts of Notch3 ICD and Hes-5 progressively decreasing in the lungs of treated rats compared to control rats (Figure 4) [14]. While placebo-treated rats developed progressive medial hyperplasia and plexiform lesions, consistent with PH in the Sugen-hypoxia model [22], rats treated with anti-NOTCH3 Ab 28042 had normal-appearing pulmonary vessels with rarely detected medial thickening, vessel occlusion, or plexiform appearance (Figure 4) [14].

Rats treated with anti-NOTCH3 Ab 28042 showed progressive diminution of RV chamber size and reversion to normal interventricular septal bowing on echocardiography over 13 weeks of treatment (Figure 4) [14]. In contrast, placebo-treated rats demonstrated no reversal of RV enlargement and developed pronounced reverse septal bowing into the left ventricle over time [14]. Pulmonary artery acceleration time (PAAT) and tricuspid annular plane systolic excursion (TAPSE) were restored to baseline values in rats treated with anti-NOTCH3 Ab 28042 (Figure 4) [14]. Additionally, rats treated with anti-NOTCH3 28042 Ab showed significant reductions in RVSP, whereas placebo-treated rats showed progressive elevation in RVSP, as measured by weekly serial invasive pressure monitoring [14]. Pulmonary angiograms performed in rats with Sugen–hypoxia-induced PH treated with the NOTCH3 28042 Ab displayed diffuse vascular blush, indicating a patent distal pulmonary vascular tree [14]. In contrast, angiograms of the control-treated rats displayed blunting of the pulmonary vasculature with an absence of peripheral filling (Figure 4) [14]. There was no significant difference in vessel/alveoli ratios between Sugen-hypoxia antibody-treated and placebo-treated rat lungs [14].

Akin to the mouse trial, no deleterious clinical side effects were detected during the duration of the experiment [14]. Microscopy revealed that in animals treated with the anti-NOTCH3 28042 Ab, vessels in organs other than the lung were morphologically normal (Figure 4) [14]. Mice and rats treated with anti-NOTCH3 Ab 28042 did not display any evidence of gastrointestinal side effects or the conversion of proliferative crypt cells to postmitotic goblet cells in intestinal villi [14], as has been reported with γ-secretase inhibitor treatment in humans and rodents [50,51]. Additionally, the complete blood counts, liver function tests, and general chemistry blood tests between treated and untreated mice and rats were not significantly different [14].

## 11. Conclusions

Clinical, biochemical, and genetic evidence indicates that vSMC proliferation in small pulmonary vessels is an essential part of the pathogenesis of PAH. Multiple studies have identified NOTCH3 as a crucial mediator of sPASMC proliferation and PAH development.

The in vitro and in vivo study of NOTCH3 signaling has informed the understanding of the molecular basis by which small precapillary pulmonary arteries develop smooth muscle hyperplasia and medial thickening, which eventually occludes the distal pulmonary arterial tree and causes clinical manifestations of PAH.

In summary:

(1) Constitutive NOTCH3 ICD expression induces sPASMC proliferation. This notion, coupled with the finding that *NOTCH3* is overexpressed at the mRNA and NOTCH3 ICD at the protein level in the lungs of humans with PAH, supports the critical role of NOTCH3 signaling in mediating sPASMC proliferation seen in this disease. Previous studies have also established a link between NOTCH3 signaling and the coordinated regulation of HES-5 effector expression in the context of sPASMC proliferation. It has been found that siRNA inhibition of *HES5* expression causes a decrease in sPASMC proliferation and a shift in gene expression in vSMCs toward a more differentiated phenotype.
(2) Human PAH vasculopathy is characterized by high steady-state levels of NOTCH3 and the downstream effector, HES-5, in vSMCs lining small precapillary pulmonary arteries. Additionally, there is a strong correlation between NOTCH3 signaling (protein levels of NOTCH3 ICD) and the magnitude of PAH in humans and PH in animals. NOTCH3 ICD protein levels in lung tissue can serve as a specific molecular marker of PAH severity in humans and PH in rodents.
(3) Notch3 signaling is required for the development of hypoxic PH in rodents. *Notch3*^−/−^ mice are resistant to the development of PH and are unable to generate a medial hyperplastic response to hypoxia because Notch3-mediated proliferative and anti-apoptotic effects on sPASMC are required for the development of pulmonary vascular medial thickening.
(4) Other forms of rodent PH, including Sugen PH, can be effectively treated by blocking Notch3 cleavage with the γ-secretase inhibitor, DAPT, or a monoclonal anti-NOTCH3-specific antibody that blocks JAG-1 binding to NOTCH3.

Collectively, these results suggest that Notch3 signaling is required for the clinical and pathologic development of PH and serves as a target for the treatment of this disease. Further studies on the NOTCH3 pathway are essential to advance the diagnosis, clinical course, and treatment of PAH. Additional validation is necessary to determine whether the NOTCH3 signaling pathway interacts with other signaling pathways at the level of downstream effector proteins. With the recent Federal Drug Administration approval of the γ-secretase inhibitor, Nirogacestat [52], for the treatment of desmoid tumors, NOTCH inhibition has become a clinical reality. However, prospective clinical trials are necessary to evaluate the efficacy and safety of targeting the NOTCH3 pathway in PAH patients through pharmacological agents or gene manipulative therapies. Longitudinal studies examining the long-term effects of targeting NOTCH3 signaling on disease progression and patient outcomes will provide valuable insights into the therapeutic potential of modulating this pathway for the successful treatment of PAH.
